# Langasite Bonding via High Temperature for Fabricating Sealed Microcavity of Pressure Sensors

**DOI:** 10.3390/mi13030479

**Published:** 2022-03-20

**Authors:** Juan Zhang, Qiulin Tan, Lei Zhang, Nan Zhao, Xiaorui Liang

**Affiliations:** 1 State Key Laboratory of Dynamic Measurement Technology, North University of China, Taiyuan 030051, China; zjtygy@163.com (J.Z.); 20210022@nuc.edu.cn (L.Z.); 15735658814@163.com (N.Z.); b1906058@st.nuc.edu.cn (X.L.); 2 Department of Mechanical Engineering, Taiyuan Institute of Technology, Taiyuan 030008, China

**Keywords:** langasite, direct bonding, high temperature, sealed microcavity, bonding mechanism

## Abstract

We proposed a novel Langasite (LGS) bonding method only using high temperature to solve the manufacturing difficulty of the sealed microcavity of pressure sensors. The optimal bonding parameters by comparative experiments were defined as 1350 °C for 3 h. Due to simple experimental conditions, low experimental cost, and be suitable for bonding wafers with various sizes, the method is convenient for popularization and mass-production, thus promoting the development of surface acoustic wave (SAW) devices at high temperatures. Simultaneously, an intact microcavity was observed by scanning electron microscopy, and a tight and void-free bonding interface with a transition layer thickness of 2.2 nm was confirmed via transmission electron microscopy. The results of tensile and leakage experiments indicated that the bonded wafer with the sealed microcavity exhibited a high bonding strength of 4.02 MPa and excellent seal performance. Compared to the original wafer, the piezoelectric constant of the LGS bonded wafer had a reduction of only 4.43%. The above characteristics show that the sealed microcavity prepared by this method satisfies the conditions for fabricating the LGS SAW pressure sensors. Additionally, based on the bonding interface characterizations, the mechanism of LGS bonding has been investigated for the first time.

## 1. Introduction

Langasite (LGS) is a promising high-temperature piezoelectric crystal, which is currently widely used in acoustic wave filters, bulk acoustic wave (BAW) sensors, and surface acoustic wave (SAW) sensors in harsh environments [1,2,3,4]. Compared to other piezoelectric crystals, such as quartz, lithium tantalate, and lithium niobate, langasite exhibits the excellent property of no phase change before melting at 1470 °C, and a lower SAW propagation velocity [5,6,7,8,9]. Therefore, LGS is considered to be suitable for fabricating the devices used in high-temperature, harsh, airtight, narrow space environments. Undoubtedly, the micromachining of LGS is usually performed to form various device structures to meet the processing requirements.

LGS belongs to the hard-brittle category of materials, which are difficult to process due to their high brittleness and low fracture toughness [10]. The micromachining technologies of LGS primarily include subtractive technology and additive technology. Wet etching [11,12], dry etching [13,14], thinning, and polishing [15,16] are the common subtractive technologies. Moreover, thin film deposition [17] and wafer bonding [18] can be classified as additive technologies. Among them, wafer direct bonding technology is a vital technique adopted for the fabrication of electronic device structure [19,20], device packaging [21,22], and the manufacture of composite substrates [23]. Compared to indirect bonding, direct bonding avoids device failure caused by mismatched thermal expansion between the wafers and the intermediate layer.

The common LGS SAW mechanical sensors primarily include pressure sensors [24,25], strain sensors [26,27], and vibration sensors. Herein, the structure of the sensors mainly featured sealed cavities, cantilever beams, and ultra-thin substrates. The formation of a sealed microcavity is one of the challenges in the preparation of SAW pressure sensors owing to the hard-brittle property of LGS. A feasible solution is to initially etch the microcavity on an LGS wafer and subsequently bond it with another intact LGS wafer to form a sealed microcavity. Therefore, the LGS direct bonding can be expected to realize the desired microstructure. However, currently, wafer bonding has been primarily developed in Si-based homogeneous or heterogeneous materials [28,29,30,31,32], and significantly less attention has been dedicated to the direct bonding of piezoelectric crystals, especially LGS. Tomita et al. presented a LiNbO_3_/LiNbO_3_ direct bonding technique via wet chemical surface activation followed by low-temperature annealing for optical waveguides [33]. Subsequently, Takigawa et al. introduced a modified surface activated bonding (SAB) method using ion beam bombardment to realize wafer bonding of SiO_2_ and LiNbO_3_ at 25 °C [34]. Xu et al. reported a low-temperature direct bonding technique involving two-step plasma activation for a LiNbO_3_/glass pair [35].

More recently, Xu et al. in our group proposed a high-temperature and high-pressure (1000 °C, 6 MPa) direct bonding method, which realized the wafer bonding of LGS for the first time [18]. However, this method required vacuum hot-pressing furnace to provide rigorous conditions such as high temperature and high pressure, so the production cost was high. Simultaneously, the bonding parameters were only applicable to the bonding of wafers with a size of 10 mm × 10 mm × 0.5 mm. For the bonding of other sizes of wafers, the pressure parameters need to be further adjusted, and too much or too little pressure may cause wafer bonding failure. Hence, the method was less versatile. Moreover, the mechanism of LGS direct bonding has not yet been clarified.

In this study, we propose an LGS bonding method only using high temperature, which greatly reduces the experimental cost and is convenient for popularization and application. Because this method only requires high-temperature environment, it does not need to adjust the bonding parameters according to the size of the wafer. Thus, it can bond all size wafers and has wider applicability. Simultaneously, the seal performance and piezoelectric property of the bonded wafer were excellent, and the tensile strength was even higher than Xu’s [18]. Furthermore, the bonding mechanism of LGS was analyzed based on bonding interface characterization for the first time, which provided a reference for the bonding of other piezoelectric crystals.

## 2. Materials and Methods

### 2.1. Sample Preparation

The LGS samples (1 cm × 1 cm × 0.05 cm in size), which were diced from 2 inches commercially available double-polished LGS crystals (0°, 138.5°, 26.7°), were used in this study. An LGS wafer with a microcavity (4 mm diameter and approximately 300 μm depth) and an intact LGS wafer comprised the bonded wafer pair. The microcavity was fabricated as follows [36]. A circular pattern with a diameter of 4 mm was firstly prepared in the center of the LGS wafer using SU8-2075 as a negative mask according to standard photolithography process. Then, the patterned wafer was wet etched in an etching solution made up of HCl and H_3_PO_4_ in a volume ratio of 1:1. The etching conditions were heating in a water bath at 80 °C for 6 h. Finally, the wafer was cleaned to remove the negative photoresist using acetone and Piranha solution (H_2_O_2_:H_2_SO_4_ = 1:3).

The LGS direct bonding process, including cleaning, plasma-activation, prebonding, and high-temperature bonding, is as follows: acetone, alcohol, and deionized water (DI) sequentially were used to clean the samples to remove any dust particles and contaminants. Subsequently, the samples were cleaned using the RCA1 solution (NH_3_H_2_O:H_2_O_2_:H_2_O = 1:2:7) in an 80 °C water bath for 15 min, followed by DI rinsing and nitrogen drying. The samples were then put in the chamber of a PVA Tepla IoN40 plasma system and activated by oxygen plasma. The activation parameters were set as follows: 200 mTorr pressure, 200 W power, 150 sccm oxygen flow rate, and 45 s [18]. After plasma activation, the samples were instantly soaked in alcohol for several seconds and brought into contact to realize their prebonding in humid air. Then, to achieve perfect prebonding, it was necessary to drive out any remaining bubbles with the aid of manual force. Finally, the samples were annealed in a muffle furnace (BLMT-1800 °C) at 1100 °C, 1200 °C, 1300 °C, and 1350 °C in ambient air for 2 h, respectively and at 1350 °C for 3 h. The heating rate was maintained at 6 °C/min.

### 2.2. Characterization

The bonding interfaces of the LGS bonded wafers were observed using scanning acoustic microscopy (C-SAM, Sonoscan D9600 C-SAM, Nordson, Westlake, OH, USA), scanning electron microscopy (SEM, JSM-7200F, JEOL, Tokyo, Japan), laser scanning confocal microscope (LSCM, LEXT OLS4100, Olympus, Shanghai, China), and transmission electron microscopy (TEM, Talos F200X, FEI, Hillsboro, OR, USA). Simultaneously, selected area electron diffraction (SAED) and energy dispersive X-ray (EDX) were also conducted to further investigate the interfacial characteristics. The bonding strength of the LGS bonded wafer was investigated using a tensile tester (Instron 2710-205) at a tensile rate of 20 mm/min. The seal performance of the bonded wafer was evaluated using a helium gas fluorine oil pressure leak detector (HF-4) and helium mass spectrometry (NHJ-400). To test whether the high-temperature bonding influenced the piezoelectric property of the wafer, the piezoelectric constants of the LGS bonded wafer were measured using a ZJ-6A quasistatic piezoelectric d_33_/d_31_ meter.

## 3. Results and Discussion

### 3.1. Bonding Interface Observation

Table 1 showed the bonding results under various bonding conditions. All the bonding failed at 1100 °C, 1200 °C, and 1300 °C for 2 h. In addition, the wafers were partially bonded at 1350 °C for 2 h and fully bonded for 3 h. Therefore, the bonding parameters were chosen as 1350 °C for 3 h. The following discussion of experimental results were carried out according to the bonded wafers using the optimal bonding parameters.

**Figure 1:**
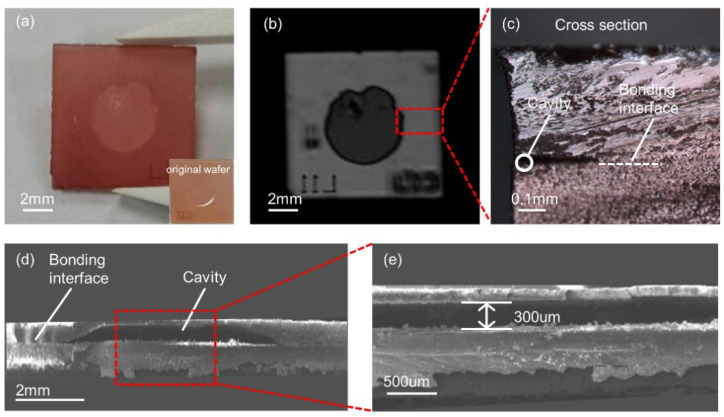
(**a**) Image of the LGS bonded wafer; (**b**) C-SAM image of the LGS bonded wafer; (**c**) LSCM image of the cross-section; (**d**) SEM image of the LGS bonded wafer; (**e**) SEM image of the microcavity.

Figure 1a depicts an image of the LGS bonded wafer, the color of which appeared darker than that of the original wafer (the inset of Figure 1a). This is because the crystal color is related to the annealing atmosphere. It was observed that the higher the oxygen content of the ambient air, the deeper was the crystal color [37]. In addition, there were some impurities on the surface, which may have been introduced during high-temperature annealing. It is necessary to further polish the surface of the bonded wafer to fabricate the devices. Furthermore, the bonding interface was observed using C-SAM and LSCM. Figure 1b depicts that the bonding interface around the cavity was clear and complete, and the cavity was well sealed. The cross-section of the bonding interface is shown in Figure 1c. The interface was tight, with no cracks observed, indicating that the LGS bonding method was feasible.

In addition, SEM analysis was performed. Figure 1d,e depict that a microcavity structure was formed in the bonding interface of the LGS bonded wafer. Moreover, the microcavity was clearly visible with no collapse, and its depth remained 300 um, which was close to the depth analyzed before the bonding. The middle of the microcavity was relatively flat, and the edge was arc-shaped. This was because the microcavity was formed by wet etching, which may lead to lateral corrosion due to the anisotropy of LGS [16].

### 3.2. TEM Analysis of Bonding Interface

**Figure 2:**
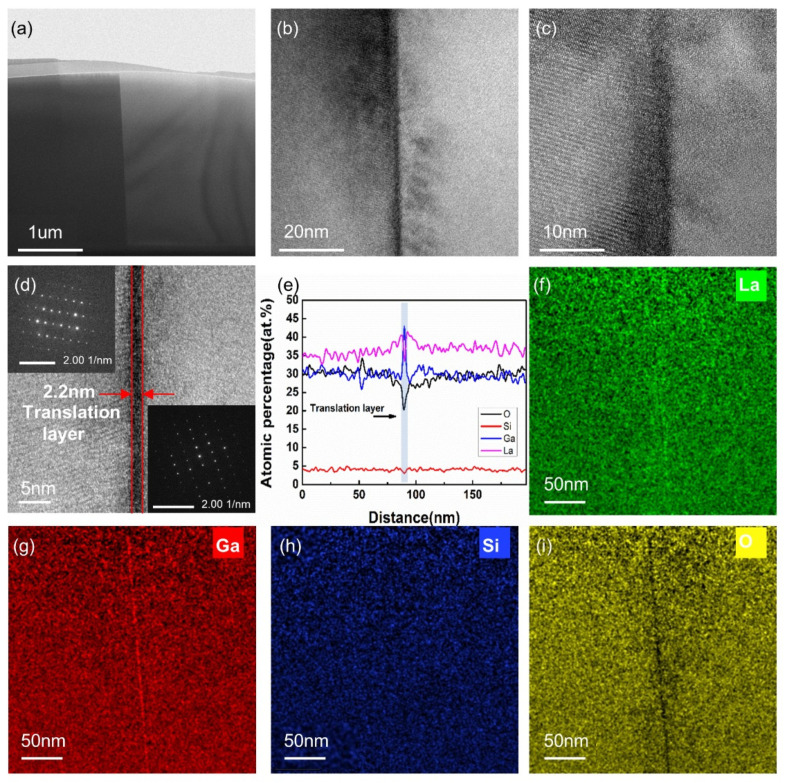
TEM images of the LGS direct bonding interface. (**a**–**d**) TEM images of the bonding interface at different magnifications; the inset images in (**d**) are EDPs of regions on both sides of the translation layer; (**e**) Elemental analysis across the bonding interface; (**f**–**i**) Elementary mappings of La, Ga, Si, and O, respectively.

To further explore the microstructure of the bonding interface, the technique of focusing ion beam (FIB) was used to fabricate the samples successfully for TEM analysis, the results of which also suggested that the bonded wafer had sufficient bonding strength. Figure 2a–d depicts TEM images of the LGS bonding interface at different magnifications. The interface with continuous lattices was smooth and compact, and periodic shading was observed at the boundary. This may be due to the periodic dislocations caused by the local strain of the crystal lattice, and the deviation of crystal orientation of the LGS samples during the bonding process might be responsible for the local strain [33]. In addition, it implied that the LGS wafers had achieved atomic-level bonding.

As depicted in Figure 2d, the LGS wafers were bonded by a transition layer with a thickness of approximately 2.2 nm. Owing to the crystalline axes unaligned by the manufacturing process errors, the electron diffraction patterns (EDPs) on both sides of the transition layer seemed to be different. However, both of them exhibited parallelogram structures, indicating that both sides of the transition layer in the bonded wafer maintained single-crystal structures. Since the transition layer was located in the middle of the bonded wafer and SAW primarily propagated along the surface of the substrate, away from the transition layer [38], the fabrication of SAW devices was hardly affected by the transition layer.

To further inspect the elemental composition at the bonding interface, elemental analyses for La, Ga, Si, and O were performed using EDX. As illustrated in Figure 2e, a continuous change in La, Ga, Si, and O occurred at the transition layer, which approximately corresponded with the thickness of the transition layer. Moreover, the distribution of the elements on both sides of the transition layer was similar, as shown in the EDX maps of La, Ga, Si, and O in Figure 2f–i, which were colored in green, red, blue, and yellow, respectively. At the transition layer, the atomic percentage of Ga increased, O and La decreased, and Si remained roughly unchanged. This result may be because Ga and O diffusion within LGS can occur at high temperatures. During the high-temperature bonding process, Ga and O in the LGS simultaneously diffused from the internal crystal to the bonding surface. Moreover, O diffusion was faster than Ga diffusion, and O further decreased as it evaporates as water according to the later bonding mechanism expressed by Equations (1)–(3), leading to Ga enrichment and O loss at the transition layer. Although the percentage of La also dropped during the transition layer, its change range was still within the normal fluctuation range of the entire test range, rather than far below the normal fluctuation range. The reason for the decrease in the proportion of La may be due to the dramatic increase in the proportion of Ga, resulting in a decrease in the proportion of La. Additionally, Si was covalently bound in the LGS, making it difficult to diffuse [39,40]. Thus, the proportion of Si remained roughly unchanged.

### 3.3. Bonding Strength

**Figure 3:**
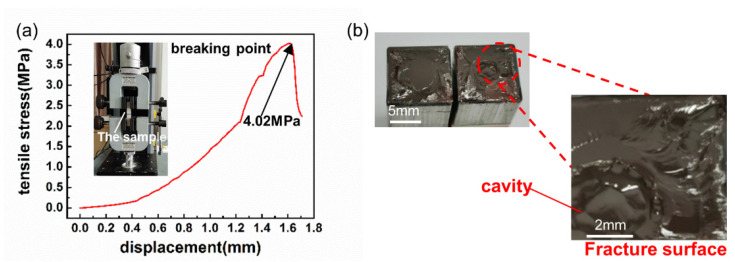
(**a**) Tensile graph of the LGS bonded wafer; (**b**) Fracture surface image of the LGS bonded wafer.

To test the bonding strength, the tensile tester was used to stretch the bonded wafer at a rate of 20 mm/min. The bonded wafer was pasted on 10 mm × 10 mm × 30 mm aluminum rods using ABS gel. As depicted in Figure 3a, the bond strength was 4.02 MPa, which satisfied the 4~5 MPa required by device manufacturing [41]. Moreover, it can be observed from Figure 3b that with the exception of the middle microcavity, all fractured sections were torn. In addition, the fracture in the bonded wafer occurred inside the crystal instead of the bonding interface, indicating that the bonding strength was approximately equal to the internal strength of the crystal. However, the tensile strength of the bonded wafer using Xu’s method was 3.81 MPa [18]. Compared to Xu’s, the tensile strength of our bonded wafer was 0.21 MPa higher than Xu’s. That is to say, under the condition of only high temperature and no high pressure, that is, the experimental conditions are simple, the bonded wafer using our method has achieved greater tensile strength.

### 3.4. Seal Performance

To examine whether the microcavity of the bonded wafer has been well sealed, a seal performance test was performed. The bonded wafer was placed into light fluorine oil at a temperature of 125 °C and a pressure of 517 kPa for 2 h. It was then put into heavy fluorine oil for 5 min, following which it was quickly removed from the helium mass spectrometer to detect the leakage rate. The results indicated no bubbles in the fluorine oil, and the leakage rate was less than the leakage limit of 5 × 10^−3^ Pa·cm^3^/s. Therefore, it was verified that the microcavity was well sealed.

### 3.5. Piezoelectric Property

The piezoelectric property of the bonded wafer was evaluated in d_33_ mode using the ZJ-6A quasistatic piezoelectric d_33_/d_31_ meter to investigate the effect of high-temperature bonding. The results shown in Table 2 indicate that the piezoelectric constant of the bonded wafer was only 4.43% lower than that of the original wafer. This measurement result suggests that both of them had almost the same characteristics, and high-temperature bonding did not severely impact the piezoelectric property of the wafer, so the bonded wafer could still be used as a substrate for the fabrication of SAW devices.

**Figure 4:**
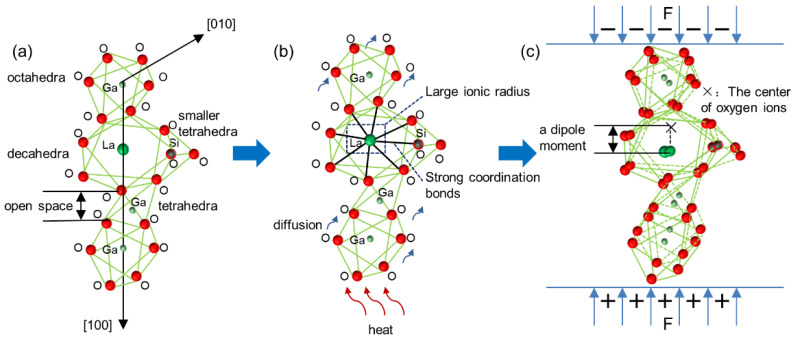
Schematic diagram of piezoelectricity mechanism of the LGS bonded wafer: (**a**) coordination polyhedral structure of LGS; (**b**) structure variation of LGS during heating; (**c**) piezoelectric effect of the LGS bonded wafer.

As shown in Figure 4a–c, in the LGS crystal structure, La and eight oxygen atoms bonded to form a decahedron [42]. The piezoelectricity mechanism of LGS can be described as follows: piezoelectricity can be generated along the [100] direction due to the structural symmetry of LGS [43,44]. When pressure was applied along the [100] direction, the position of La^3+^ remained unchanged because of the repulsion force from Ga^3+^, and the center of oxygen ions largely shifted in the [−100] direction due to the open space as a damper. Thus, the centers of the cations and anions did not overlap and produced a dipole moment, resulting in the generation of piezoelectricity. The larger the distance of the centers of the cations and anions, the stronger is the piezoelectric property.

As the crystal structure of the LGS bonded wafer virtually remained the same, as indicated by the TEM results, the bonded wafer retained its piezoelectricity. Furthermore, the size of the ionic radius in decahedrons affected the piezoelectricity [45,46,47]. The decahedron with a larger ionic radius had more space to compress, making the distance between the centers of oxygen ions and cations larger, and resulting in a higher piezoelectricity. During the high-temperature bonding process, on the one hand, La has a large ionic radius, which makes it difficult to diffuse at high temperatures, thus leading to the high stability of the decahedron [39]. On the other hand, the La–O mean bond length is 2.5935 Å [48], which is approximately equal to the sum of La^3+^ ionic radius and O^2-^ ionic radius 2.58 Å. (The ionic radius of La^3+^ is 1.16 Å, and the ionic radius of O^2−^ is 1.42 Å, according to eight coordination [49,50].) This indicates that La^3+^ and O^2−^ formed strong coordination bonds using the hybrid orbital, which made it difficult to modify the La–O bond length at high temperatures. Hence, the size of the decahedron had a small variation, and the piezoelectric property of the bonded wafer slightly declined.

### 3.6. Bonding Mechanism

**Figure 5:**
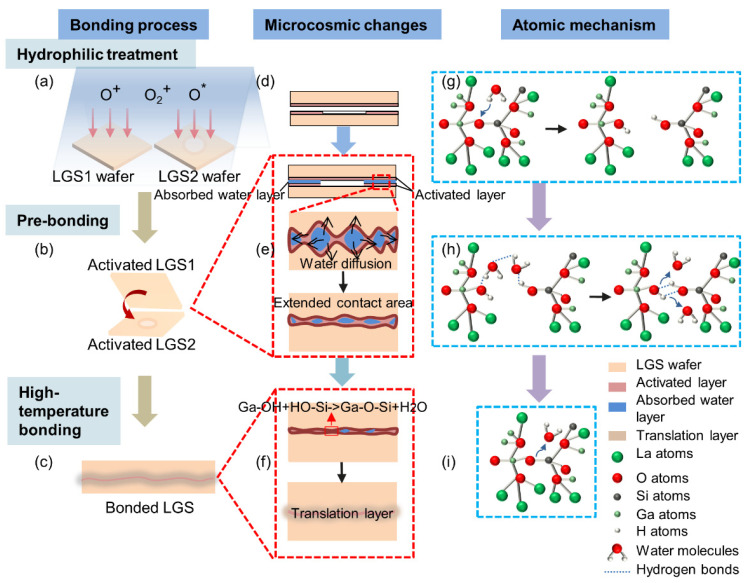
Schematic diagram of the mechanism for high-temperature direct bonding of LGS via O_2_ plasma activation: (**a**–**c**) bonding process; (**d**–**f**) microcosmic changes of bonding interface; (**g**–**i**) atomic mechanism of bonding.

The mechanism of LGS direct bonding could be elucidated based on the bonding interface characterization. Figure 5 illustrates the schematic diagram of the mechanism for high-temperature direct bonding of LGS via O_2_ plasma activation. The bonding process primarily includes hydrophilic treatment, prebonding, and high-temperature bonding, as shown in Figure 5a–c.

In the hydrophilic treatment, O_2_ plasma treatment, on the one hand, can decrease the surface roughness of the samples by clearing away the contaminants. On the other hand, it can form a surface-activated layer, which allows the surface to absorb more hydroxyl groups and further enhance the surface hydrophilicity [51]. The mechanism of hydrophilicity may be as follows: according to the literature [52], water molecules include a hydrogen atom as a proton donor and an oxygen atom providing a lone pair of electron orbitals. The H–O bond in the water molecule has almost the same length as the Si–O bond in SiO_2_ so that proton transfer can occur, resulting in the fracture of the Si–O–Si bond and the establishment of Si–OH. Similarly, the crystal structure of LGS encompasses eight-fold coordinated La ions, octahedrally coordinated Ga ions, and tetrahedrally coordinated Ga and Si ions [6]. The bond length of Ga–O may be 1.719 Å, 1.748 Å, 1.810 Å, 1.908 Å, and 1.994 Å according to the different sites of Ga and the bond length of Si–O is 1.719 Å, both of which are approximately equivalent to the H–O bond length in water molecules [48]. Therefore, LGS may undergo proton transfer with water molecules, leading to bond breakage and hydroxyl adsorption. As shown in Figure 5g, taking Ga–O–Si in LGS as an example, the oxygen atom adsorbs water molecules due to the hydrogen bond. Subsequently, the H atom in the water molecule transfers to the oxygen atom in Ga–O–Si. Simultaneously, the oxygen atom in the water molecule provides electrons to Ga/Si atoms, thereby causing the rupture of Ga–O–Si bonds and the formation of Ga–OH and Si–OH. Similarly, hydroxylation may occur in Ga–O–Ga and Si–O–Si, thus forming Ga–OH and Si–OH.

After hydrophilic treatment, the activated LGS surfaces were brought into contact with each other at 25 °C, and in the prebonding interface, the hydrogen bonds with a weak bonding strength were formed (Figure 5h). As time passed, the water molecules between the two LGS wafers evaporated along with the prebonding interface to the surrounding environment and through the activated surface into the LGS bulk (Figure 5e). At high temperatures, the accelerated diffusion of water molecules considerably reduces the distance between –OH groups on the surface. Dehydroxylation reactions occurred among Si–OH and Ga–OH groups according to Equations (1)–(3). Thus, strong bonds of Ga–O–Si, Ga–O–Ga, and Si–O–Si were formed owing to the irreversibility of the dehydration reaction above 400 °C (Figure 5f,i) [53]. Moreover, the activated layer induced by O_2_ plasma treatment had a porous structure which enhanced the diffusivity of the water and further accelerated the chemical reactions of Equations (1)–(3) [54]. Additionally, the activated layer may begin to soften and close these gaps by the viscous flow of the material at high temperatures, thus increasing the bond strength [55]. Therefore, the strong bonding of LGS can be achieved.

(1) $$\mathrm{Ga}\text{-}\mathrm{OH}+\mathrm{HO}\text{-}\mathrm{Si}\to \mathrm{Ga}\text{-}O\text{-}\mathrm{Si}+H_{2}O$$

(2) $$\mathrm{Ga}\text{-}\mathrm{OH}+\mathrm{HO}\text{-}\mathrm{Ga}\to \mathrm{Ga}\text{-}O\text{-}\mathrm{Ga}+H_{2}O$$

(3) $$\mathrm{Si}\text{-}\mathrm{OH}+\mathrm{HO}\text{-}\mathrm{Si}\to \mathrm{Si}\text{-}O\text{-}\mathrm{Si}+H_{2}O$$

## 4. Conclusions

In this study, a bonding method only using high temperature (1350 °C, 3 h) to achieve LGS sealed microcavity was proposed. The higher bonding temperature makes the molecular motion more intensely and accelerates the dehydration reactions to form a strong bonding layer without high pressure. The bonding strength of 4.02 MPa was obtained for the LGS bonded wafer, which is considered to be sufficient for the fabrication of devices. Simultaneously, the microcavity of the structure was found to remain intact and well-sealed based on SEM observations and results of leakage experiments. Furthermore, the LGS bonded wafer exhibited excellent piezoelectric property, demonstrating that the plasma-activated and high-temperature direct bonding method is appropriate to fabricate the LGS SAW devices. In addition, TEM observations revealed that the bonding interface was uniform and seamless, thereby achieving LGS bonding at the atomic scale. Consequently, the mechanisms of O_2_ plasma-activated high-temperature direct bonding were established for the first time. On the one hand, O_2_ plasma activation removed organic contaminants and reduced the roughness of the surface. On the other hand, it enabled the formation of a porous activated layer on LGS surfaces, which not only increased its superficial area, resulting in the absorption of more –OH groups, but also simultaneously acted as a reservoir for water molecules during the bonding procedure, leading to the continuous occurrence of dehydroxylation reactions and formation of strong bonds. Moreover, high temperatures may cause a viscous flow of the material, which can close interfacial gaps, thereby achieving the strong bonding of LGS.

Compared to current high-temperature and high-pressure bonding technology, this method has low experimental cost, thus leading to be easy to popularize. Simultaneously, it is suitable for bonding wafers with various sizes. In conclusion, the LGS direct bonding method including O_2_ plasma-activation and high-temperature annealing shows tremendous potential for SAW applications at high temperatures, and the analysis of the bonding mechanism of LGS can also provide a reference for the bonding of other piezoelectric crystals.

## Figures and Tables

**Table 1:** The bonding results under various bonding conditions.

Bonding Conditions		Bonding Results
Temperature (°C)	Time (h)	
1100	2	Failure
1200	2	Failure
1300	2	Failure
1350	2	Partially bonded
1350	3	Fully bonded

**Table 2:** Comparison of the piezoelectric properties of the original and bonded wafers.

Wafer Type	Piezoelectric Constant (|d|/pC/N)				Decrease (%)
	1	2	3	Average	
Original wafer	0.7	0.8	0.8	0.767	-
Bonded wafer	0.8	0.7	0.7	0.733	4.43%

## Data Availability

The data has been included in the text.
